# From two-dimensional graphene oxide to three-dimensional honeycomb-like Ni_3_S_2_@graphene oxide composite: insight into structure and electrocatalytic properties

**DOI:** 10.1098/rsos.171409

**Published:** 2017-12-20

**Authors:** Xinting Wei, Yueqiang Li, Wenli Xu, Kaixuan Zhang, Jie Yin, Shaozhen Shi, Jiazhen Wei, Fangfang Di, Junxue Guo, Can Wang, Chaofan Chu, Ning Sui, Baoli Chen, Yingtian Zhang, Hongguo Hao, Xianxi Zhang, Jinsheng Zhao, Huawei Zhou, Shuhao Wang

**Affiliations:** 1 School of Chemistry and Chemical Engineering; College of Materials Science and Engineering; Shandong Provincial Key Laboratory of Chemical Energy Storage and Novel Cell Technology, Liaocheng University, Liaocheng 252000, People's Republic of China; 2 Liaocheng Seismic Hydrochemistry Station, Liaocheng, People's Republic of China; 3 College of Materials Science and Engineering, Qingdao University of Science and Technology, Qingdao 266042, People's Republic of China

**Keywords:** three-dimensional Ni_3_S_2_@ graphene oxide, catalysts, structure, electrocatalytic properties

## Abstract

Three-dimensional (3D) graphene composites have drawn increasing attention in energy storage/conversion applications due to their unique structures and properties. Herein, we synthesized 3D honeycomb-like Ni_3_S_2_@graphene oxide composite (3D honeycomb-like Ni_3_S_2_@GO) by a one-pot hydrothermal method. We found that positive charges of Ni^2+^ and negative charges of NO_3_^−^ in Ni(NO_3_)_2_ induced a transformation of graphene oxide with smooth surface into graphene oxide with wrinkled surface (w-GO). The w-GO in the mixing solution of Ni(NO_3_)_2_/thioacetamide/H_2_O evolved into 3D honeycomb-like Ni_3_S_2_@GO in solvothermal process. The GO effectively inhibited the aggregation of Ni_3_S_2_ nanoparticles. Photoelectrochemical cells based on 3D Ni_3_S_2_@GO synthesized at 60 mM l^−1^ Ni(NO_3_)_2_ exhibited the best energy conversion efficiency. 3D Ni_3_S_2_@GO had smaller charge transfer resistance and larger exchange current density than pure Ni_3_S_2_ for iodine reduction reaction. The cyclic stability of 3D honeycomb-like Ni_3_S_2_@GO was good in the iodine electrolyte. Results are of great interest for fundamental research and practical applications of 3D GO and its composites in solar water-splitting, artificial photoelectrochemical cells, electrocatalysts and Li-S or Na-S batteries.

## 1. Introduction

Graphene is composed of SP^2^ hybrid C atoms. The thickness of two-dimensional (2D) monolayer graphene is 3.35 Å. Six C atoms in the same plane bond with the adjacent C atoms in the sigma format, which makes the graphene have good structural rigidity. The orbit of surplus p is perpendicular to the graphene plane. The big *π* bonds are formed by overlapping of other p orbits. The electrons in big *π* bonds can move freely, which gives good conductivity to graphene. The unique monolayer graphene has a large theoretical specific surface area of about 2630 m^2^ g^−1^. It also has high conductivity, high electron mobility (15 000 cm^2^ V^−1^ s^−1^) [1], and thermal conductivity, quantum Holzer effect, quantum tunnelling effect [2], super mechanical properties [3], and so on. However, it lacks semiconductor properties, which limits its application in many fields.

Three-dimensional (3D) graphene composites have drawn increasing attention in energy storage/conversion applications due to their unique structures and properties [4–9]. Freestanding, lightweight 3D graphene networks as ultralight and flexible supercapacitor electrodes were prepared from pressed Ni foam [10]. A 3D graphene-based hierarchically porous carbon has been prepared by a dual template strategy and explored as an electrode for capacitive deionization [11]. A 3D carbon fibre/reduced graphene oxide composite textile was prepared by introducing 2D reduced graphene oxide interfaces into one-dimensional (1D) carbon fibre networks [12].

The composites of graphene and transition metal complexes (TMC) possess electrical conductivity, thermal conductivity, structure stability and excellent catalytic activity. The structures of TMC/graphene composite material have four categories: 0D/2D (zero-dimensional TMC/two-dimensional graphene), 1D/2D (one-dimensional TMC/two-dimensional graphene), 2D/2D (two-dimensional TMC/two-dimensional graphene) and 3D (TMC/three-dimensional graphene).

3D NiS/G composite and 3D CoS/G composite were prepared by three steps: growing graphene by chemical vapour deposition, coating precursors and further annealing [13]. The dye-sensitized solar cells (DSCs) with 3D NiS/G composite and 3D CoS/G composite counter electrodes showed good electrocatalytic activity and photovoltaic conversion efficiencies of 5.04% and 5.25%, respectively. We previously reported that unique ZnS nanobuns decorated with 2D reduced graphene oxide were synthesized using one-pot solvothermal method. Graphene in ZnS@GO remained in 2D structures (flake-like shape with few wrinkles). Herein, we synthesized 3D honeycomb-like Ni_3_S_2_@graphene oxide composite (3D honeycomb-like Ni_3_S_2_@GO) by a one-pot hydrothermal method. We study the structural characteristics of 3D honeycomb-like Ni_3_S_2_@GO evolved from 2D graphene oxide. The electrocatalytic characteristics of 3D honeycomb-like Ni_3_S_2_@GO for iodine reduction reaction are investigated by electrochemical impedance (EIS) and Tafel polarization. The effects of 3D honeycomb-like Ni_3_S_2_@GO structure on photovoltaic parameters are also investigated.

## 2. Material and methods

### 2.1. Preparation of pure Ni_3_S_2_ block

Typically, 0.0017 mol Ni(NO_3_)_2_·6H_2_O was dissolved in 30 ml deionized water by vigorous agitation. Thioacetamide (TAA) (0.023 mol) was dissolved in 30 ml deionized water by vigorous agitation. The above two solutions were mixed. The mixture was stirred for 0.5 h at room temperature and then transferred into a Teflon-lined autoclave. After being heated at 200°C for 24 h, the product was cooled to room temperature naturally. The product was washed three times with water and ethanol.

### 2.2. Preparation of three-dimensional honeycomb-like Ni_3_S_2_@graphene oxide

Specific synthesis method of GO could be seen in our previous literature [14–16]. GO (1.125 g 1 wt%) was dispersed in 10 ml deionized water by ultrasound. 0.0017 mol Ni(NO_3_)_2_·6H_2_O was dissolved in 30 ml deionized water by vigorous agitation. TAA (0.023 mol) was dissolved in 20 ml deionized water by vigorous agitation. The above three solutions were mixed. The mixture was stirred for 0.5 h at room temperature and then transferred into a Teflon-lined autoclave. After being heated at 200°C for 24 h, the product was cooled to room temperature naturally. The product was washed three times with water and ethanol.

### 2.3. Photoanode preparation and cell fabrication

A 12 µm thick layer was deposited on fluorine-doped tin oxide glass by printing 20 nm-sized TiO_2_ particles (P25, Degussa, Germany) [15,17]. The obtained film was sintered at 500°C. After cooling to 90°C, the TiO_2_ films were immersed in a solution of N719 dye (5 × 10^−4^ M) in acetonitrile/*tert*-butyl alcohol (1 : 1 volume ratio) for 20 h. The triiodide/iodide electrolyte for cell testing includes LiI (0.03 M), 1-butyl-3-methylimidazolium iodide (0.6 M), I_2_ (0.03 M), 4-*tert*-butylpyridine (0.5 M), guanidinium thiocyanate in acetonitrile (0.1 M). DSCs were assembled by a TiO_2_ photoanode with the corresponding counter electrode sandwiching the redox couple in the electrolyte. Symmetrical cells with an effective area of 0.64 cm^2^ were analysed by a Tafel-polarization test and by EIS experiments.

### 2.4. Characterization

To analyse as-synthesized composite electrocatalyst, X-ray diffraction (XRD) patterns were acquired using a PANalytical X'Pert diffractometer (Cu K*α* radiation at *λ* = 1.5406 Å) sampling at 5° min^−1^, 36 kV and 20 mA. As-prepared micro- or nanostructures were characterized and analysed by scanning electron microscopy (SEM; Nova Nano SEM 450). The photocurrent–voltage performance of DSCs with 0.16 cm^2^ photoanode film was measured without metal mask by a Keithley digital source meter (Keithley 2400, USA) equipped with a solar simulator (IV5, PV Measurements, Inc., USA). EIS and Tafel experiments were done with symmetrical electrodes in the dark using an electrochemical workstation (CHI760 Chenhua, China). Cyclic voltammetry (CV) was performed in a three-electrode configuration. The triiodide/iodide electrolyte for CV testing includes LiI (2 mM), LiClO_4_ (20 mM) and I_2_ (0.2 mM).

## 3. Results and discussion

**Figure 1.**
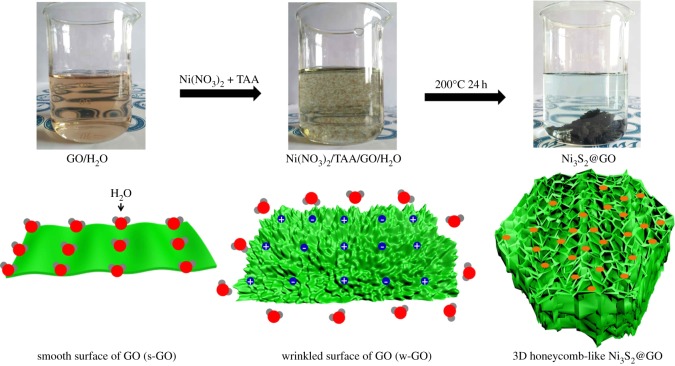
Picture and structural evolution of 3D honeycomb-like Ni_3_S_2_@GO from graphene oxide with smooth surface and graphene oxide with wrinkled surface.

The graphene oxide prepared by the Hummers method usually contains a large number of functional groups. The functional groups (hydroxyl and carboxyl groups) on the graphene surface can make graphene more compact and stable when graphene is combined with other materials. As shown in figure 1, graphene oxide prepared by us can be well dispersed in H_2_O to form a homogeneous dispersion. The graphene oxide in H_2_O (GO/H_2_O) can be stable for several months or longer without delamination. The reason for this is the strong hydrogen bonding between hydroxyl and carboxyl groups on the surface of graphene oxide and H_2_O. Therefore, the surface of the graphene oxide in H_2_O is relatively smooth, rather than wrinkled. The graphene oxide with a smooth surface is s-GO. When TAA was added into GO/H_2_O, no obvious change was observed in the homogeneous dispersion. However, when Ni(NO_3_)_2_ solution or mixed solution of Ni(NO_3_)_2_ and TAA were added into GO/H_2_O, homogeneous dispersion shows lots of flocculent graphene oxide immediately, as shown in figure 1. Because lots of positive charges of Ni^2+^ and negative charges of NO_3_^−^ in Ni(NO_3_)_2_ destroy the hydrogen bonding between graphene oxide and H_2_O, the surface of flocculent graphene oxide should be wrinkled rather than smooth, as shown in figure 1. The graphene oxides with wrinkled surface were named w-GO. SEM of w-GO is shown in electronic supplementary material, figure S1. w-GO has two advantages: on the one hand, it can keep the 2D structure of graphene; on the other hand, the wrinkles on the surface can increase the physical and chemical properties in graphene devices. The w-GO suspension is transferred into a Teflon-lined autoclave. After crystallizing and cross-linking of w-GO suspension at 200°C for 24 h, 3D honeycomb-like structures of Ni_3_S_2_@GO were formed, as shown in figure 1.

**Figure 2.**
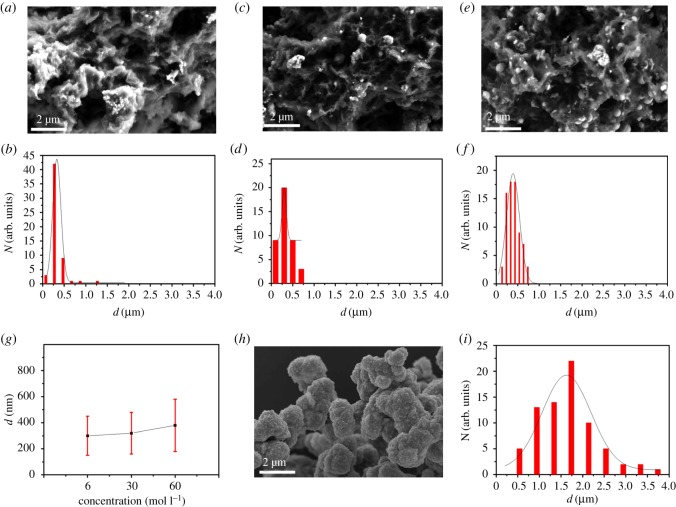
(*a,b*) SEM image and size distribution for 3D honeycomb-like Ni_3_S_2_@GO synthesized under 6 mM l^−1^ Ni(NO_3_)_2_; (*c,d*) SEM image and size distribution for 3D honeycomb-like Ni_3_S_2_@GO synthesized under 30 mM l^−1^ Ni(NO_3_)_2_; (*e*,*f*) SEM image and size distribution for 3D honeycomb-like Ni_3_S_2_@GO synthesized under 60 mM l^−1^ Ni(NO_3_)_2_; (*g*) the relationship between the size of Ni_3_S_2_ on graphene surface and the concentration of Ni(NO_3_)_2_; (*h,i*) SEM image and size distribution for pure Ni_3_S_2_ synthesized under 30 mM l^−1^ Ni(NO_3_)_2_.

We investigated the effect of different concentrations of Ni(NO_3_)_2_ on the morphology of Ni_3_S_2_@GO. As can be seen from figure 2, the microstructures of all the synthesized Ni_3_S_2_@GO samples obtained using 6, 30 and 60 mM l^−1^ Ni(NO_3_)_2_ have 3D honeycomb-like structures. This kind of 3D honeycomb-like structure possessed larger specific surface area and more surface catalytic activity sites. In addition, the coupling between the walls of w-GO and Ni_3_S_2_ will play the role of synergistic catalysis. There are few Ni_3_S_2_ nanoparticles on the 3D honeycomb-like Ni_3_S_2_@GO under 6 mM l^−1^ Ni(NO_3_)_2_. The size of Ni_3_S_2_ nanoparticles on the 3D honeycomb-like Ni_3_S_2_@GO is approximately 300 nm (figure 2*a,b*). When the concentration of Ni(NO_3_)_2_ is 30 mM l^−1^, the numbers of Ni_3_S_2_ nanoparticles on the 3D honeycomb-like Ni_3_S_2_@GO are increased. The size of Ni_3_S_2_ nanoparticles on the 3D honeycomb-like Ni_3_S_2_@GO is approximately 320 nm, as shown in figure 2*c,d*. The numbers of the Ni_3_S_2_ nanoparticles on the 3D honeycomb-like Ni_3_S_2_@GO under 60 mM l^−1^ are more than those under 6 and 30 mM l^−1^ Ni(NO_3_)_2_. The size of Ni_3_S_2_ nanoparticles on the 3D honeycomb-like Ni_3_S_2_@GO is approximately 380 nm (figure 2*e,f*). As shown in figure 2*g*, the size of graphene surface Ni_3_S_2_ is gradually increased with increasing concentration of Ni(NO_3_)_2_. The SEM results of pure Ni_3_S_2_ material synthesized under 30 mM l^−1^ Ni(NO_3_)_2_ are shown in figure 2*h*. It can be seen that the pure Ni_3_S_2_ block was larger than Ni_3_S_2_ in Ni_3_S_2_@GO. The size of Ni_3_S_2_ block is approximately 1.7 µm, as shown in figure 2*i*. The results suggested that the presence of graphene significantly inhibits aggregation of Ni_3_S_2_ nanoparticles, which is consistent with our previous findings [11].

**Figure 3.**
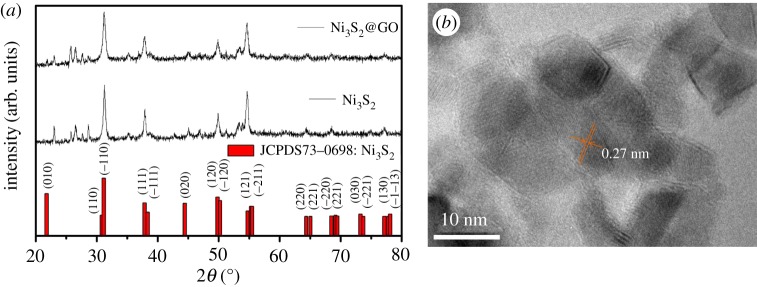
(*a*) XRD patterns of as-prepared 3D honeycomb-like Ni_3_S_2_@GO and pure Ni_3_S_2_ synthesized under 30 mM l^−1^ Ni(NO_3_)_2_; (*b*) high-resolution transmission electron micrograph of as-prepared 3D honeycomb-like Ni_3_S_2_@GO under 30 mM l^−1^ Ni(NO_3_)_2_.

The XRD pattern of the 3D honeycomb-like Ni_3_S_2_@GO powder is shown in figure 3*a*. In order to classify the diffraction peaks, we synthesized pure Ni_3_S_2_ as the contrast. The diffraction pattern of 3D honeycomb-like Ni_3_S_2_@GO is consistent with that of pure Ni_3_S_2_. All of the peaks are in accordance with the Ni_3_S_2_ standard card (PDF#73-0698). The strong diffraction peaks at 31.26° and 54.7° corresponded to (−110) and (−211) crystal planes. The other diffraction peaks at 23.04°, 37.94° and 49.88° corresponded to (010), (111), (120) crystal planes. From further structural analysis by high-resolution transmission electron microscopy, the lattice spacing of Ni_3_S_2_ on Ni_3_S_2_@GO is 0.27 nm, which is close to [−110] spacing of Ni_3_S_2_ (PDF#73-0698).

**Figure 4.**
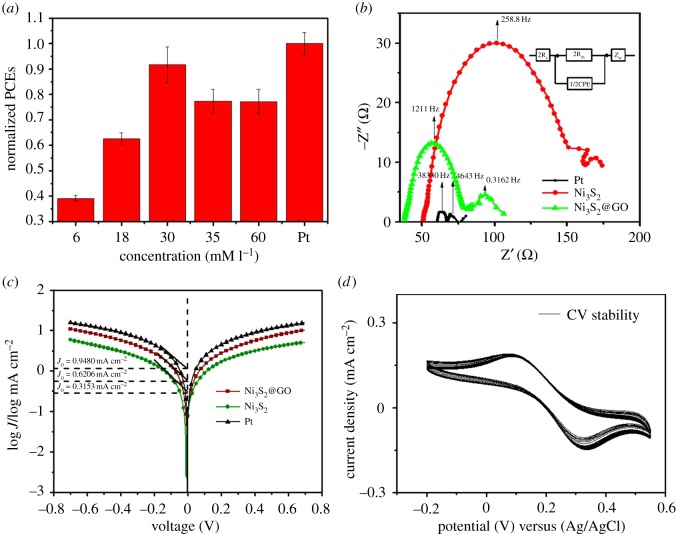
(*a*) The normalized power conversion efficiency (PCE) based on 3D honeycomb-like Ni_3_S_2_@GO synthesized by different concentration of Ni(NO_3_)_2_; (*b*) EIS of symmetrical cells fabricated with two identical 3D honeycomb-like Ni_3_S_2_@GO or pure Ni_3_S_2_ (synthesized by 30 mM l^−1^ Ni(NO_3_)_2_) under the bias voltage with open voltage corresponding to photovoltaic devices; (*c*) Tafel polarization curves of symmetrical cells fabricated with two identical 3D honeycomb-like Ni_3_S_2_@GO or pure Ni_3_S_2_ (synthesized by 30 mM l^−1^ Ni(NO_3_)_2_) under the bias voltage with open voltage corresponding to photovoltaic devices; (*d*) the cyclic stability of 3D honeycomb-like Ni_3_S_2_@GO (synthesized by 30 mM l^−1^ Ni(NO_3_)_2_) in the iodine electrolyte.

3D honeycomb-like Ni_3_S_2_@GO synthesized by different concentrations of Ni(NO_3_)_2_ was prepared in thin film by the spraying method and used as a counter electrode in DSCs. Meanwhile, pyrolytic platinum was prepared to be used as a reference. The normalized power conversion efficiency is shown in figure 4*a*. The results indicated that the highest energy conversion efficiency is based on 3D honeycomb-like Ni_3_S_2_@GO synthesized by 30 mM l^−1^ Ni(NO_3_)_2_. The effect of 3D honeycomb-like Ni_3_S_2_@GO structure synthesized by different Ni(NO_3_)_2_ concentration on open circuit voltage (*V*_oc_), short circuit current density (*J*_sc_) and fill factor is shown in electronic supplementary material, figures S2, S3 and S4. The best energy conversion efficiency based on 3D Ni_3_S_2_@GO synthesized at 60 mM l^−1^ Ni(NO_3_)_2_ is shown in electronic supplementary material, figure S5. To investigate the reason for the good performance of 3D honeycomb-like Ni_3_S_2_@GO materials in DSCs, EIS and Tafel polarization were carried out. EIS is an electrochemical method widely used for the characterization of counter electrode. Figure 4*b* is a typical Nyquist diagram. Each Nyquist diagram usually consists of two semicircles. The resistance of left semicircle starting on *X*-axis represented the series resistance (*R*_s_). The value of left semicircle diameter represented charge transfer resistance (*R*_ct_) between the electrode and electrolyte. *R*_ct_ occurs in the high-frequency region and is closely related to the electrocatalytic properties. According to the symmetrical cell equivalent circuit diagram in figure 4*b*, the EIS parameters obtained by Z-View software are listed in table 1.

**Table 1.** Series resistance (*R*_s_), charge transfer resistance (*R*_ct_) and exchange current density based on the symmetrical cells of 3D honeycomb-like Ni_3_S_2_@GO, Ni_3_S_2_ (synthesized by 30 mM l^−1^ Ni(NO_3_)_2_) and Pt in the iodine electrolyte.

electrolytes	CEs	*R*_s_/*Ω*	*R*_ct_/*Ω*	*J*_0_/mA cm^2^
I^−^/I_3_^−^	Ni_3_S_2_@GO	38.19	40.29	0.6206
	Ni_3_S_2_	50.72	106.4	0.3153
	Pt	60.86	6.866	0.9480

The values of *R*_s_ for 3D honeycomb-like Ni_3_S_2_@GO and pure Ni_3_S_2_ in I^−^/I_3_^−^ electrolyte system are 38.19 Ω and 50.72 Ω, respectively. The main reason for the smaller *R*_s_ of 3D honeycomb-like Ni_3_S_2_@GO is the high conductivity of graphene in composites. The value of *R*_ct_ for 3D honeycomb-like Ni_3_S_2_@GO is 40.29 Ω, which is much smaller than that of pure Ni_3_S_2_ (106.4 Ω). The results indicated that 3D honeycomb-like Ni_3_S_2_@GO exhibited better electrocatalytic activity than pure Ni_3_S_2_ for the reduction reaction of iodine. The frequency at highest point of left semicircle for Ni_3_S_2_ is 258.8 Hz, which corresponds to a time constant of 0.003864 s. The frequency at highest point of left semicircle for 3D honeycomb-like Ni_3_S_2_@GO is 1211 Hz, which corresponds to a time constant of 0.0008921 s. The time constants also indicated that the catalytic performance of 3D honeycomb-like Ni_3_S_2_@GO was better than that of pure Ni_3_S_2_. *R*_ct_ and the exchange current density (*J*_0_) in Tafel was inversely proportional, according to *R*_ct_ = *RT/nFJ*_0_. The values of *J*_0_ for 3D honeycomb-like Ni_3_S_2_@GO and pure Ni_3_S_2_ are 0.6317 mA cm^−2^ and 0.36625 mA cm^−2^, respectively. The result indicated that the iodine reduction reaction has faster electron exchange on the surface of 3D honeycomb-like Ni_3_S_2_@GO, which is consistent with the EIS results. The cyclic stability of 3D honeycomb-like Ni_3_S_2_@GO in the iodine electrolyte is shown in figure 4*d*. The current density and the potential position had no significant change, which indicated the good stability of 3D honeycomb-like Ni_3_S_2_@GO in the iodine electrolyte.

## 4. Conclusion

In sum, 3D honeycomb-like Ni_3_S_2_@GO was synthesized by a one-pot hydrothermal method. The positive charges of Ni^2+^ and negative charges of NO_3_^−^ in Ni(NO_3_)_2_ induced a transformation of s-GO into w-GO. The GO can effectively inhibit the aggregation of Ni_3_S_2_ nanoparticles. 3D honeycomb-like Ni_3_S_2_@GO exhibited good electrocatalytic activity and photoelectrochemical performance. These findings are of great interest for fundamental research and practical applications of 3D graphene oxides and their composites.

## Supplementary Material

Electronic Supplementary Information

## Data accessibility

Additional data are in the electronic supplementary material.
