# Supplementary material for: From two-dimensional graphene oxide to three-dimensional honeycomb-like Ni3S2@graphene oxide composite: insight into structure and electrocatalytic properties
Source: R Soc Open Sci. 2017 Dec 20;4(12):171409. doi: 10.1098/rsos.171409 (PMC5750029; doi:10.1098/rsos.171409)
Supplement: Electronic Supplementary Information [file rsos171409supp1.docx]

***Electronic Supplementary Information***

From Two-dimensional Graphene Oxide to Three-dimensional Honeycomb-like Ni_3_S_2_@Graphene Oxide Composite: Insight into Structure and Electrocatalytic Properties

Xinting Wei ^a^, Yueqiang Li ^a^,Wenli Xu ^a^, Kaixuan Zhang ^a^, Jie Yin ^a,*^, Shaozhen Shi ^a^, Jiazhen Wei ^a^, Fangfang Di ^a^, Junxue Guo ^a^, Can Wang ^a^, Chaofan Chu ^a^, Ning Sui ^b^, Baoli Chen ^a^, Yingtian Zhang ^a^, Hongguo Hao ^a^, Xianxi Zhang ^a^, Jinsheng Zhao ^a^, Huawei Zhou ^a, *^ Shuhao Wang ^a^

*^a^ School of Chemistry and Chemical Engineering; College of Materials Science and Engineering; Liaocheng University, Liaocheng 252000, China. Shandong Provincial Key Laboratory of Chemical Energy Storage and Novel Cell Technology*

*^b^* Liaocheng Seismic Hydrochemistry Station, China.

*^c^ College of Materials Science and Engineering, Qingdao University of Science and Technology, Qingdao 266042, China*

** Corresponding authors:* [*zhouhuaweiopv@163.com*](mailto:zhouhuaweiopv@163.com)

[*yinjieily@163.com*](mailto:yinjieily@163.com)


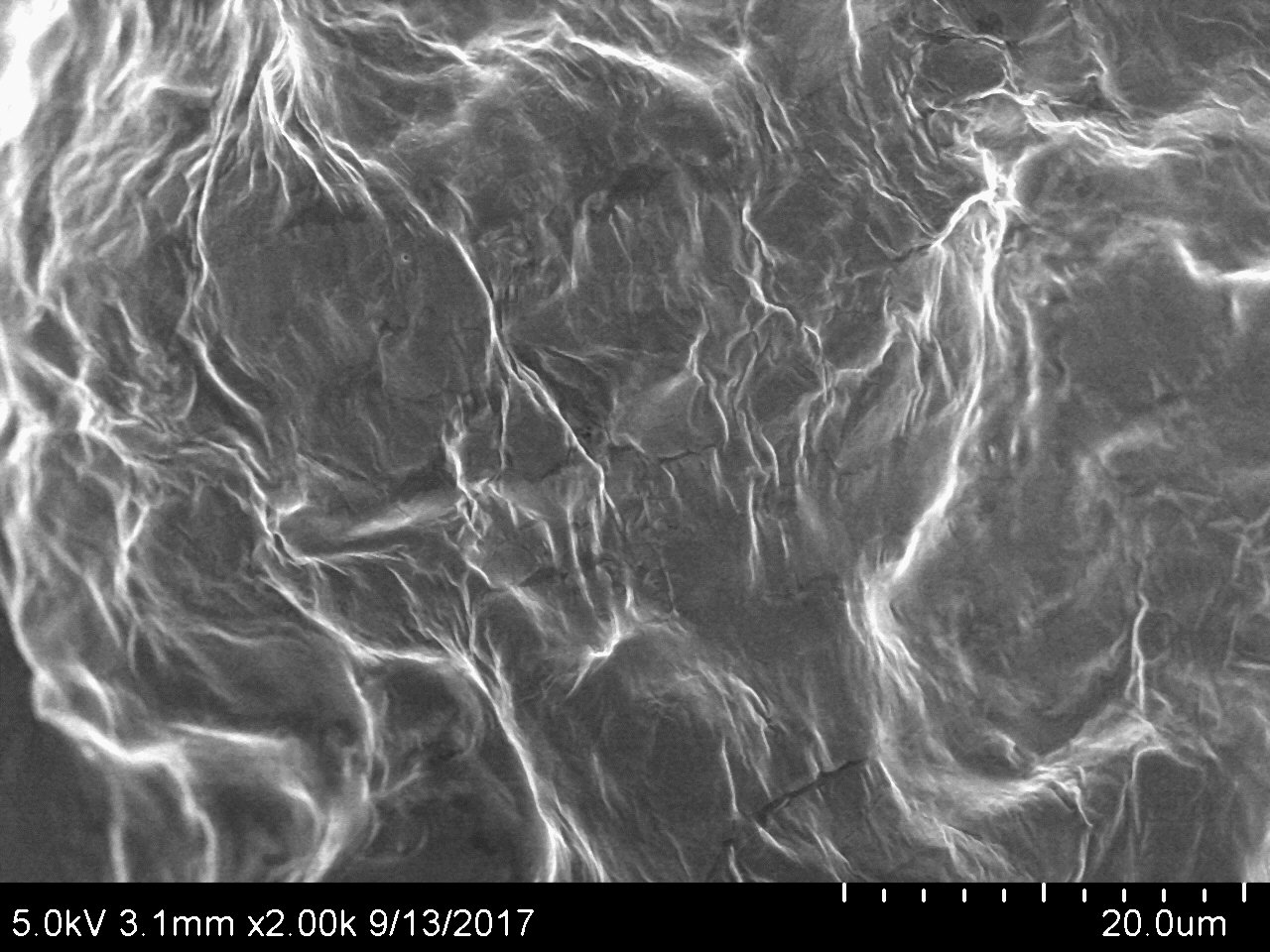


Figure S1 SEM of w-GO

**Figure S2.** The normalized open circuit voltage based on 3D honeycomb-like Ni_3_S_2_@GO synthetized by different concentration of Ni(NO_3_)_2_

**Figure S3.** The normalized short circuit current density based on 3D honeycomb-like Ni_3_S_2_@GO synthetized by different concentration of Ni(NO_3_)_2_

**Figure S4.** The normalized fill factor based on 3D honeycomb-like Ni_3_S_2_@GO synthetized by different concentration of Ni(NO_3_)_2_

**Figure S5** The best energy conversion efficiency based on 3D Ni_3_S_2_@GO synthetized at 60 mM L^-1^ Ni(NO_3_)_2_
